# Unique Photoactivated Time‐Resolved Response in 2D GeS for Selective Detection of Volatile Organic Compounds

**DOI:** 10.1002/advs.202205458

**Published:** 2023-01-19

**Authors:** Mohammad Reza Mohammadzadeh, Amirhossein Hasani, Keyvan Jaferzadeh, Mirette Fawzy, Thushani De Silva, Amin Abnavi, Ribwar Ahmadi, Hamidreza Ghanbari, Abdelrahman Askar, Fahmid Kabir, R.K.N.D. Rajapakse, Michael M. Adachi

**Affiliations:** ^1^ School of Engineering Science Simon Fraser University Burnaby British Columbia V5A 1S6 Canada; ^2^ Department of Computer Science and Software Engineering Concordia University Montreal Quebec H3G 1M8 Canada; ^3^ Department of Physics Simon Fraser University Burnaby British Columbia V5A 1S6 Canada

**Keywords:** 2D Materials, GeS, machine learning, sensors, volatile organic compounds (VOCs) detection

## Abstract

Volatile organic compounds (VOCs) sensors have a broad range of applications including healthcare, process control, and air quality analysis. There are a variety of techniques for detecting VOCs such as optical, acoustic, electrochemical, and chemiresistive sensors. However, existing commercial VOC detectors have drawbacks such as high cost, large size, or lack of selectivity. Herein, a new sensing mechanism is demonstrated based on surface interactions between VOC and UV‐excited 2D germanium sulfide (GeS), which provides an effective solution to distinguish VOCs. The GeS sensor shows a unique time‐resolved electrical response to different VOC species, facilitating identification and qualitative measurement of VOCs. Moreover, machine learning is utilized to distinguish VOC species from their dynamic response via visualization with high accuracy. The proposed approach demonstrates the potential of 2D GeS as a promising candidate for selective miniature VOCs sensors in critical applications such as non‐invasive diagnosis of diseases and health monitoring.

## Graphical abstract

Here, a new sensing mechanism based on surface interactions between volatile organic compounds (VOCs) and UV‐excited 2D germanium sulfide is demonstrated. The sensor exhibits a unique dynamic response to each VOC during illumination which can be considered as “fingerprints” for VOC identification. Machine learning is utilized to distinguish VOC species from their dynamic response via visualization with high accuracy.

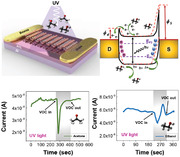

## 1 Introduction

Volatile organic compounds (VOCs) are organic compounds that evaporate at room temperature and pressure due to high vapor pressure.^[^ ^1^, ^2^ ^]^ Recent research has shown that VOCs can act as a biomarker in human breath, blood, sweat, urine, or other biofluid for non‐invasive diagnosis of numerous diseases.^[^ ^3^, ^4^, ^5^ ^]^ Low concentrations of VOCs are produced via human metabolism, which can penetrate into blood circulation and be exhaled through pulmonary ventilation.^[^ ^6^ ^]^ For example, existence of low concentrations of acetone, ethanol, butanone, toluene, and styrene in exhaled breath are correlated with esophagogastric cancer, gastric cancer, peptic ulcer disease, oral squamous cell carcinoma, and Alzheimer's disease, respectively.^[5,^ ^7^ ^]^ Detection of VOCs can be used as a screening tool to detect diseases in the early stage when treatment options are more effective.^[^ ^8^ ^]^ Furthermore, there are various sources of VOC emissions such as petroleum fuels, industrial solvents, household products, pesticides, building materials, and burning fossil fuels.^[^ ^9^, ^10^ ^]^ Exposure to VOCs can be very harmful to humans, inducing various health conditions and diseases such as cancers, skin irritation, impairment of the nervous system, and lung damage.^[^ ^11^, ^12^ ^]^ Therefore, a portable sensor that can rapidly identify and quantify VOCs can be an effective tool for remote health monitoring and diagnostic applications.

There are a number of methods that can identify and quantify VOCs including gas chromatography‐mass spectrometry, infrared spectrometry, and magnetic resonance.^[^ ^13^, ^14^, ^15^, ^16^, ^17^, ^18^, ^19^ ^]^ However, these methods have drawbacks such as high cost and complexity, large size, and require regular maintenance and training to operate.^[^ ^11^ ^]^ Commercial portable VOC detection methods include electrochemical, nondispersive IR, and chemiresistive metal oxide detectors.^[^ ^20^, ^21^, ^22^ ^]^ Drawbacks of these methods include need for cartridge replacements due to catalyst exhaustion, detection limited to a specific VOC, or limited ability to discriminate one VOC from another or interfering compounds.^[^ ^5^ ^]^ Lack of selectivity in existing portable sensor technologies is a concern due to generation of false positives in health applications. Recently, Zhu et al. proposed a new approach for highly selective VOC detection based on plasma‐assisted mid‐IR and machine learning.^[^ ^11^ ^]^ A machine‐learning tool was used to visualize and identify VOCs from mid‐IR absorption spectra enhanced from plasma.^[^ ^11^ ^]^ However, this method requires specific conditions such as a plasma which leads to high cost and complexity. Therefore, a new low‐cost technique that can identify VOCs is required for health applications.

2D materials such as transition metal dichalcogenides (TMDs) and graphene have gained interest for sensing applications due to high carrier mobility and high sensitivity at room temperature arising from their high surface to volume ratio.^[^ ^23^, ^24^, ^25^ ^]^ Group‐IV monochalcogenides which have a MX chemical formula (M: Sn, Ge; X: Se, and S) are another family of 2D materials with a puckered honeycomb structure similar to phosphorene. Unlike phosphorene, group‐IV monochalcogenides MX cannot be easily oxidized due to strong polar chemical bonding.^[^ ^26^ ^]^ Therefore, the electronic properties of 2D monochalcogenides MX can be relatively preserved in ambient conditions.

2D Germanium Sulfide (GeS) is an oxide‐resistance material from the 2D monochalcogenide MX family, which has a high carrier mobility of 3680 cm^2^ V^−1^s^−1^, a large bandgap of 1.6 eV, high photoresponse, piezoelectric properties, and tunable band structure by external modulation.^[^ ^26^, ^27^ ^]^ Density functional theory (DFT) studies have shown that VOC molecules are physisorbed on GeS surface via weak van der walls interaction, leading to a high rate of charge transfer and enhanced thermodynamic stability and sensitivity.^[^ ^26^ ^]^ Furthermore, Ulaganathan et al. fabricated a broadband photodetector based on multi‐layered GeS with superior photoresponse, high stability, and fast response.^[^ ^28^ ^]^ These unique features suggest that 2D GeS could be a remarkable candidate for high‐performance photo‐assisted VOC detection.

In recent years, studies have shown that the dynamic response contains valuable information such as response time and recovery time that may not be available in static measurements.^[^ ^29^, ^30^, ^31^, ^32^, ^33^, ^34^, ^35^, ^36^ ^]^ Herein, for the first time, we demonstrate a new technique to produce a unique time‐resolved electrical response to different VOCs. Mechanically exfoliated 2D GeS crystal electrically contacted by interdigitated Cr/Au electrodes exhibited a unique time‐resolved electrical response to six different VOCs measured assisted by UV photoexcitation. The unique time‐resolved fingerprint of each VOC enables the discriminative detection of VOCs via visualization using a machine learning algorithm. The results indicate a new low‐cost mechanism of VOC discrimination using photo‐excited 2D GeS for potential non‐invasive diagnostic applications such as human breath analysis. The scheme of time‐resolved photo‐excited sensing also has the potential to be applied to a broad range of sensing platforms beyond VOC detection such as industrial gas sensors and biosensors.

## 2 Results and Discussion

**Figure 1:**
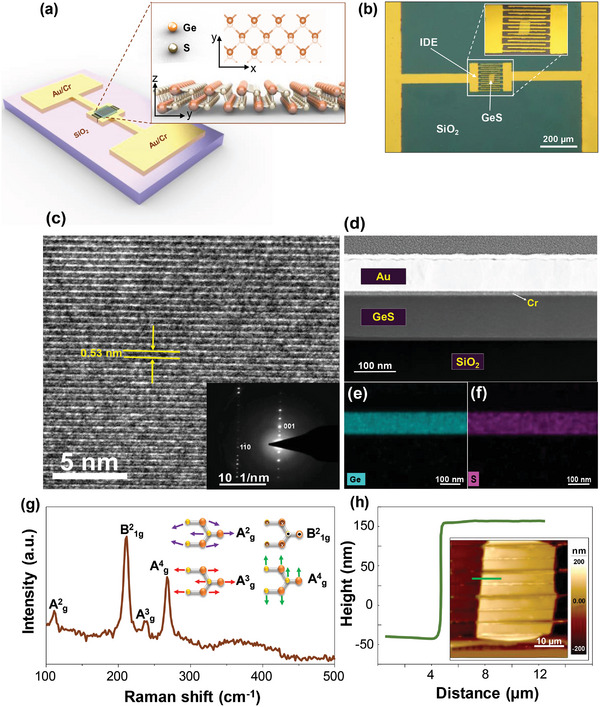
GeS VOC sensor and characterization of GeS a) Schematic illustration of GeS VOC sensor and zoomed‐in illustration of the GeS crystal structure; b) top‐view optical microscopy image of the fabricated GeS sensor; c) cross sectional HR‐TEM and SAED images (inset), d) cross sectional HAADF‐STEM image, e) elemental mapping images of Ge, f) S, g) Raman spectrum of the GeS crystal; h) AFM height profile and height map (inset) of the GeS flake.

The active layer of the sensor is multilayer 2D GeS crystals, which have a puckered honeycomb lattice structure (**Figure** **1**a). A top‐view optical microscopy image of the GeS sensor is depicted in Figure 1b. The sensor was fabricated on a 300 nm thick thermal oxide (SiO_2_) coated silicon wafer (resistivity ≤ 0.005 Ω‐cm). Interdigitated electrodes (IDE) with an electrode spacing of 10 µm and a width of 500 nm made of chromium/gold (10/60 nm in thickness) are used as electrical contacts to the GeS crystal. GeS has a distorted orthorhombic structure belonging to the Pcmn‐$D_{2h}^{16}$ space group^,[^ ^37^, ^38^ ^]^ and can be viewed as a derivative of the orthorhombic black phosphorus. GeS exhibits *p*‐type semiconductor behavior with an indirect bandgap of ≈1.6 eV in the bulk form.^[^ ^27^, ^39^, ^40^ ^]^ The cross sectional high‐resolution TEM (HR‐TEM) image of GeS crystal indicates a lattice fringe spacing of 5.3 Å (Figure 1c) which is in agreement with the results reported previously.^[^ ^41^ ^]^ The inset of Figure 1c shows the selected area electron diffraction (SAED) of GeS crystal which corresponds to orthorhombic crystal structure oriented along the [110] zone axis.^[^ ^42^ ^]^ Figure 1d shows the cross sectional high‐angle annular dark‐field scanning transmission electron microscopy (HAADF‐STEM) image for GeS sensor device. The energy dispersive X‐ray spectroscopy (EDS) elemental mappings of the GeS sensor show that the interfaces are homogenous and no oxidation was detected in the GeS crystal (Figure S1, Supporting Information; Figure 1e,f). Raman spectroscopy measurements of the flake indicate the four Raman modes of $A_{g}^{2}$, $B_{1g}^{2}$, $A_{g}^{3}$, and $A_{g}^{4}$ at 113.1, 213.6, 238.1, and 271.2 cm^−1^, respectively (Figure 1g), which agrees with Raman spectra reported in other layered GeS studies.^[^ ^37^, ^43^, ^44^ ^]^ The observed *A_g_* and *B_3g_* in‐plane phonons are associated with shear modes in which the adjacent layers are vibrating parallel with respect to each other in the armchair and zigzag directions, respectively.^[^ ^37^, ^43^ ^]^ The GeS flake thickness was measured to be ≈170 nm based on Atomic Force Microscopy (AFM) height profile (Figure 1h), corresponding to ≈320 number of layers.^[^ ^45^ ^]^

### 2.1 VOC Sensing Properties Under Dark Conditions

**Figure 2:**
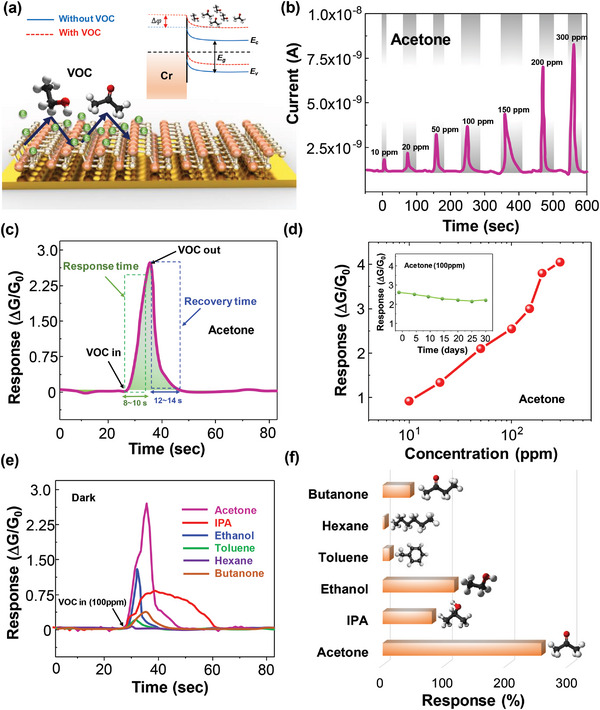
Sensor response to VOCs under dark conditions. a) Schematic diagram of VOC adsorption on GeS surface under dark conditions and energy band diagram of Au/Cr and GeS interface; b) Time‐resolved sensor response (Δ*G*/*G_o_*) of the GeS sensor when exposed to acetone at different concentrations under dark environment; c) enlarged sensor response showing the response time (8–10 s) and recovery times (12–14 s) of the GeS sensor to 100 ppm of acetone; d) The sensor response as a function of acetone concentrations from 10 to 300 ppm under dark condition and (inset) the long‐term stability; e) one cycle of sensing time‐resolved response of the GeS sensor toward six different VOC species including ethanol, 2‐propanol, acetone, hexane, toluene, and butanone at 100 ppm concentration under dark environment; and f) Amplitude of sensor response under dark environment to different VOCs each at 100 ppm.

The VOC sensing characteristics of the GeS sensor were first measured at room temperature (25 °C) without light illumination. Adsorption of VOC molecules causes the Fermi level of *p*‐type GeS to shift toward the valence band resulting in a decrease in Schottky barrier height (*ϕ*) and an increase in conductivity^[^ ^46^ ^]^ (**Figure** **2**a). The adsorbed VOC molecules also provide extra electrons to GeS and increase the conductance (G) of GeS when the VOC is introduced. The change in Schottky barrier height and carrier concentration result in a change in conductance (ΔG) of the GeSfrom its original conductance before VOC (G_0_). The Fermi level moves toward the valance or conduction band, due to adsorption and desorption between VOC molecules and GeS surface. The time‐resolved current response of the GeS sensor when exposed to acetone at different concentrations (10, 20, 50, 100, 150, 200, and 300 ppm (parts per million)) is shown in Figure 2b. The time‐resolved current responses for other VOCs, including 2‐propanol (IPA), ethanol, toluene, hexane, and butanone, introduced at different concentrations are shown in Figure S2 (Supporting Information). The time‐resolved sensor response (ΔG/G_0_) to acetone at a concentration of 100 ppm exhibited a fast response time of 8.6 s and recovery time of 13.4 s (Figure 2c). The sensor response as a function of acetone concentrations from 10 to 300 ppm at room temperature is shown in Figure 2d. In addition, the long‐term stability of GeS sensor was examined upon exposure to 100 ppm acetone for 30 days (inset of Figure 2d). The sensor response remained ≈2.35 (or 235%) within the duration of 30 days, suggesting good long‐term stability of the GeS sensor. The GeS sensors were exposed to different VOCs to investigate the selectivity characteristics of sensors. The sensor response toward 100 ppm of ethanol, acetone, 2‐propanol, toluene, hexane, and butanone is shown in Figure 2e.

Due to the difference in coefficient of diffusion for each VOC, the response and recovery time of each VOC differs from one another. Despite the difference in response amplitude, the sensor's time‐resolved response to the different VOCs has similar shapes making distinguishing VOCs from one another difficult. This lack of discrimination is a common challenge for many VOC sensors. The response of the VOC sensor during exposure to the six different VOCs at 100 ppm concentration under dark conditions is shown in Figure 2f. The sensor exhibited the highest response to acetone compared to the other VOCs due to the higher absorption energy of acetone compared with other VOCs, which is in agreement with DFT simulation results reported in the literature.^[^ ^26^ ^]^

### 2.2 Photoactivated Signatures by UV‐light Irradiation

**Figure 3:**
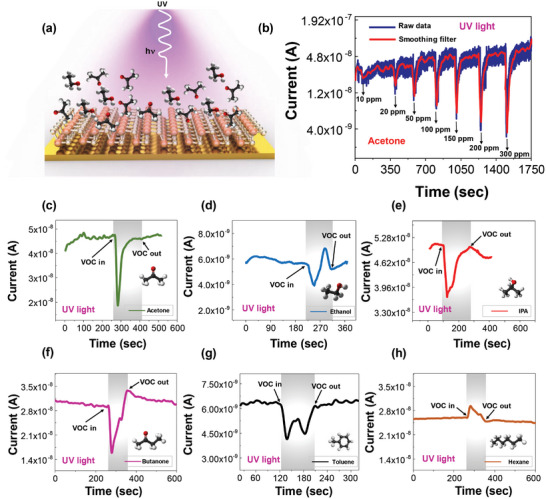
UV‐enhanced VOC detection a) Schematic diagram of VOC adsorption on the GeS surface under UV‐light illumination; b) time‐resolved current of the GeS sensor when exposed to different concentrations of acetone under UV‐light illumination; Time‐resolved current of the GeS sensor to 200 ppm of c) acetone, d) ethanol, e) 2‐propanol, f) butanone, g) toluene and h) hexane under UV illumination.

The GeS sensors were excited with UV light with a peak wavelength of *λ* = 365 nm through a quartz window of the characterization chamber during exposure to VOCs, illustrated in **Figure** **3**a, schematically. The time‐resolved current response for acetone at different concentrations (10, 20, 50, 100, 150, 200, and 300 ppm) under UV‐light illumination is shown in Figure 3b. The blue curve is the raw data and the red curve is the smoothing filtered curve used for reducing noise. Under UV light irradiation the sensor exhibited a response in an opposite direction (downward) than under dark conditions (upward as shown in Figure 2b) when exposed to acetone.

There are several possible explanations for this phenomenon: 1) the absorption of acetone molecules induced by UV light is more favorable than the interaction between UV photons and GeS surface.^[^ ^47^ ^]^ 2) electron‐photon interaction and UV‐light absorption can be significantly promoted via acetyl groups which are oxidizing agents.^[^ ^47^ ^]^ 3) Absorption of UV photons in UV wavelength range from 225 to 320 nm by acetone molecules. This behavior also can be found in other literatures which used 2D materials as sensing layers.^,[^ ^48^, ^49^, ^50^, ^51^ ^]^

The GeS sensor also shows a unique time‐resolved current response to six different VOCs tested: ethanol, acetone, 2‐propanol, toluene, hexane, and butanone (Figure 3c–h). Each VOC was introduced at the same concentration of 200 ppm.

When the GeS sensor is exposed to a VOC during UV illumination, there is a competition between the photogeneration of free carriers in *p*‐type GeS by UV light and capturing of electrons by VOCs on the GeS surface. This change is ascribed to lower hole carrier concentrations in GeS due to the recombination of electrons with holes from photogenerated hole‐electrons pairs. There is an electron‐photon interaction within VOC molecules, contributing to a transient behavior consisting of both oxidizing and reducing processes over time.^[^ ^51^ ^]^ For example, when the sensor is exposed to ethanol, the current decreases initially, followed by an increase in current, after which it stabilizes to a steady‐state value (Figure 3d). These unique dynamic responses can be considered as “fingerprints” for VOC identification. Figure S3 (Supporting Information) indicates the time‐resolved response of the GeS sensor for six VOCs at different concentrations (50, 100, 150, and 200 ppm), showing consistent VOC “fingerprints” (with different amplitudes) at different concentrations. To the best of the authors' knowledge, the photo‐excited GeS sensor is the first solid‐state device to exhibit a unique shape in the time‐resolved electrical response to six different VOCs. The unique response to different was observed only in GeS under UV‐light illumination. Other 2D materials including UV‐illuminated MoS_2_ and MoSe_2_, and GeS without UV‐light illumination did not generate unique electrical response shapes to the different VOCs and instead produced time‐dependent responses with a similar shape but different amplitude. Figure S4a,b (Supporting Information) shows three cycles of time‐resolved sensor response of the UV‐illuminated MoS_2_ sensor to ethanol and 2‐propanol at a concentration of 200 ppm under UV‐light illumination. Similarly, the time‐resolved sensor response of UV‐illuminated MoSe_2_ to ethanol and 2‐propanol are shown in Figure S4c,d (Supporting Information), respectively. The MoS_2_ and MoSe_2_ sensors’ time‐dependent responses to the two VOCs consisted of one positive peak per VOC cycle, exhibiting a similar shape, making discriminating the VOCs from one another difficult. The unique VOC responses observed in GeS are attributed to dopant‐type tunability by VOCs and significant photoresponse behavior.^[^ ^26^ ^]^

### 2.3 Machine Learning‐Assisted VOC Recognition

**Figure 4:**
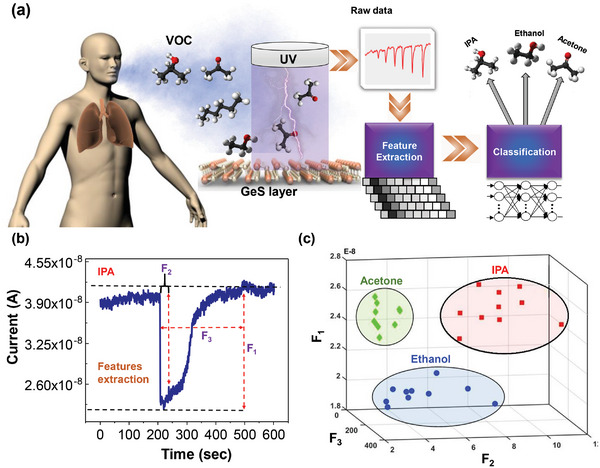
Mechanism of VOC sensing. a) Illustration of the classification of VOCs during breath analysis based on electrical output data acquired from the GeS sensor and machine‐learning assisted feature extraction, b) F_1_, F_2_, and F_3_ features extraction from the time‐resolved electrical response from the GeS sensor, and c) 3D distribution of the three features for the VOC data points. The data points for each VOC are clustered together (Acetone in green, 2‐propanol in red, and ethanol in blue) which can be used for VOC identification.

To assess the feasibility of the VOC sensor for non‐invasive breath analysis for diagnostic applications, a classification analysis with a machine‐learning approach was performed, as illustrated in **Figure** **4**a. In the first step, three features (F_1_, F_2_, and F_3_) were extracted from the raw data, which consisted of a sample set of 10 measurements for each VOC. F_1_ corresponds to the change in current between the steady‐state signal before the VOC is introduced and the peak current amplitude after the VOC is introduced. F_2_ is the time difference between the initial signal spike caused by the introduction of VOC and the next time the signal flattens out. Finally, F_3_ is the change in current between the initial signal spike when the VOC is introduced and the steady‐state current after the VOC is no longer present. A graphical representation of the F_1_, F_2_, and F_3_ are shown in Figure 4b. The 3D plot of the three features for all VOC data points is shown in Figure 4c.

Exhaled breath contains a mixture of gases (nitrogen, oxygen, carbon dioxide, etc.), water vapor, and various VOCs. Therefore, there is a need to identify VOCs in the presence of other VOCs. Figure S5 (Supporting Information) shows that the GeS sensor produces a relatively unique time‐resolved response when exposed to two mixtures of VOCs (ethanol and 2‐propanol vs ethanol, 2‐propanol, and acetone), which are different from those of the individual VOCs on their own (Figure 3c,d,e). After recording multiple mixed signals, feature extraction and classification were applied again using machine learning. A significant correlation (Pearson Correlation coefficient with alpha value = 0.05) between the two mixed VOCs (ethanol and 2‐propanol), and three mixed VOCs (ethanol, 2‐propanol, and acetone) were observed, suggesting that the time‐resolved GeS VOC sensor could be used to distinguish VOC species in mixtures.

**Figure 5:**
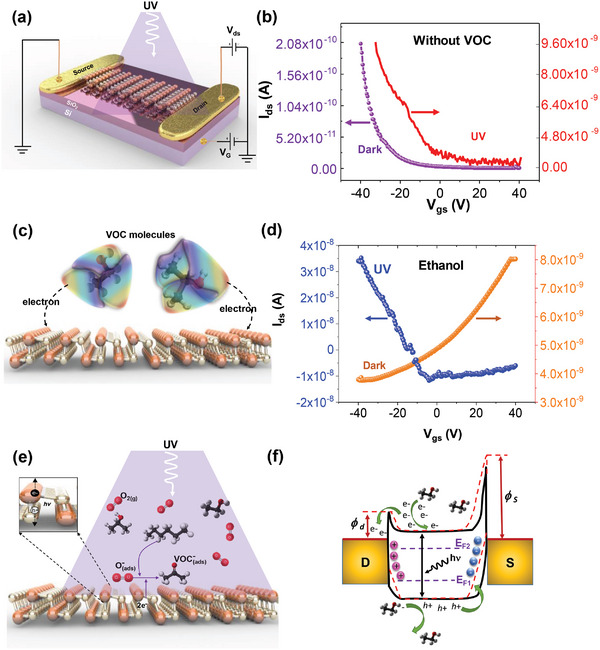
Mechanism of VOC sensing. a) Schematic illustration of a GeS FET under UV‐light irradiation; b) transfer curve (*I*_ds_ vs *V*_gs_) of the GeS FET shows *p*‐type semiconducting behavior under both dark and UV illumination conditions in the absence of VOCs. c) Schematic diagram of charge transfer between VOC molecules and GeS surface; d) transfer curve of the GeS FET during ethanol exposure shows *n*‐type behavior under dark conditions but maintains *p*‐type behavior under UV excitation. e) Schematic illustration of the VOCs sensing mechanism of GeS sensor under UV illumination; and f) energy band diagram of GeS FET sensor.

To further investigate the VOC sensing mechanism, a GeS field‐effect transistor (FET) was fabricated to investigate the transport characteristics of the GeS crystal during exposure to UV illumination and VOCs. **Figure** **5**a illustrates the schematic of the GeS FET. The transfer curves (drain‐source current *I*_ds_ vs gate‐source voltage *V*_gs_) of GeS FET under dark and UV‐light illumination are shown in Figure 5b. The gate voltage (*V*_gs_) was swept from −40 to 40 V while keeping the drain‐source voltage (*V*_ds_) bias constant at 0.5 V. The shape of the transfer curve indicates *p*‐type semiconducting behavior in the GeS under both dark and UV illumination conditions. A shift in threshold voltage (*V*_th_) and enhancement in current amplitude is observed under UV illumination due to the hot generation of carriers. The carrier density near the GeS surface can be modified by the physisorption of VOC molecules and charge transfer between VOC and GeS (Figure 5c). Figure 5d shows the transfer curve of the GeS FET remeasured under dark and UV illumination conditions while the sensor is exposed to a continuous flow of ≈1000 ppm ethanol. The GeS FET transfer curve shows *p*‐type semiconducting behavior in presence of ethanol under UV illumination. The photogenerated electrons recombine with some holes and reduce the number of carriers (holes), but the majority of carriers remained holes. However, in the presence of ethanol and the dark condition, the GeS FET switches to *n*‐type behavior (Figure 5d orange curve). The changing of doping behavior from *p*‐ to *n*‐type in the presence of ethanol reveals that the exposure to VOCs under dark conditions can change the majority carrier type along the GeS surface from holes to electrons. Similar dependency of the majority dopant type on light conditions (*n*‐type behavior under dark conditions, and *p*‐type behavior under UV illumination) was also observed when the GeS FET was exposed to 2‐propanol and acetone (Figure S6, Supporting Information).

The time‐resolved GeS sensor electrical response to VOCs is also dependent on the UV‐light intensity. The time‐resolved sensor response to ethanol and 2‐propanol as a function of light intensity is shown in Figures S7 and S8 (Supporting Information). The shape of the time‐resolved electrical response varies due to changes in photogenerated hole‐electron pair concentration at the GeS surface as a function of incident UV‐light intensity. When UV illumination was simultaneously imposed, photogenerated electrons were attracted to the GeS surface due to the inward built‐in electric field. Then more electrons participated in the reaction with VOC molecules, adsorbed O_2_ (ad) and O_2_(hv), leading to a larger response compared to that in dark conditions (see Figure 5e).^[^ ^52^ ^]^ Figure 5f illustrates the energy band diagram of the GeS FET. There is a Schottky barrier contact between Au/Cr electrodes and GeS nanosheet. The GeS‐Cr Schottky barrier height at the source (*ϕ*_s_) is higher than the GeS‐Cr Schottky Barrier at the drain (*ϕ*_d_). Exposing the GeS to VOC molecules changes the semiconducting behavior of GeS from *p*‐ to *n*‐type. During exposure of VOCs to the GeS, the VOC molecules also act as electron donors at the GeS surface, resulting in an increase in conductance.^[^ ^53^ ^]^

### 2.4 Environmental Effect and Stability Test

**Figure 6:**
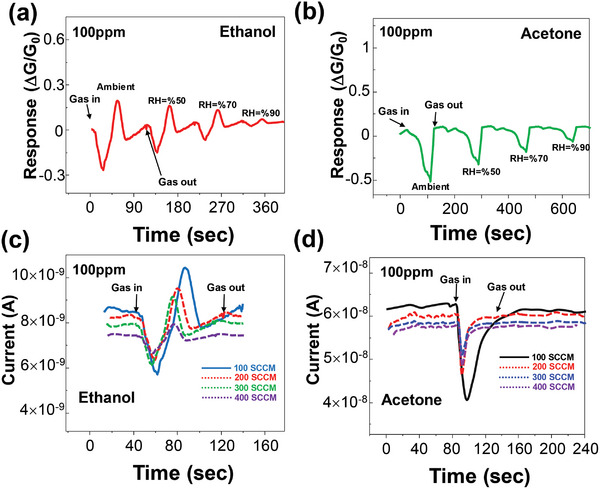
Sensor response to environmental conditions. Effect of the relative humidity on the time‐resolved response of the GeS sensor toward a) 100 ppm of ethanol, and b) acetone. c) Effect of the N_2_ flow rate on the time‐resolved response of the GeS sensor to d) 100 ppm of ethanol, and e) acetone.

Furthermore, the effect of environmental conditions on dynamic response was examined. **Figure** **6**a,b show 100 ppm of ethanol and acetone sensing behavior, respectively of the GeS sensor under UV light illumination for relative humidity (RH) varying from 50 to 90%, respectively. The sensor output current amplitude decreases for both VOCs for increasing RH. Importantly, the unique time‐dependent shape of the ethanol and acetone are preserved, demonstrating that discrimination of these VOCs can be maintained for different levels of RH. Figure 6c,d indicates the time‐resolved response of the GeS sensor to 100 ppm of ethanol and acetone, respectively, under UV light illumination with the variation of N_2_ (carrier gas) flow rate. As a result, response and recovery times become faster as the N_2_ flow rate increases. The sensor's amplitude or sensitivity decreased as the N_2_ flow rate increased, which can be explained by a variation in the effective concentration due to the kinetic transport of the VOC‐carrying flow.^[^ ^54^, ^55^ ^]^ Again, the unique shape of the time‐dependent current response to ethanol and acetone is preserved for different flow rates.

Gas sensing experiments were also performed at different temperatures, 25 °C (room temperature), 42, 73, and 125 °C, when exposed to acetone and 2‐propanol each at a fixed concentration of 100 ppm to investigate the effect of temperature on the device performance (Figure S9, Supporting Information). The amplitude of the current response decreases at elevated temperatures compared to room temperature, while the recovery time improves by thermal excitation. Although temperature variation altered the relative response, the shape of the time‐dependent response, i.e., the signature, remains unique to each VOC. Elevated thermal energy generally accelerates the desorption process of VOC molecules leading to lower recovery time.^[^ ^56^, ^57^ ^]^ The carrier concentration and hence conductivity increases due to thermal annealing, which lowers the relative response.^[^ ^58^ ^]^

These above measurements taken at different relative humidity, flow rate, and temperature demonstrate that although the amplitude and response times are a function of surrounding conditions, the shape of the dynamic response remains unique to each VOC tested.

To ensure the consistency of sensitive material without breakage, four different sensor devices were tested when exposed to ethanol at a flow rate of 200 ppm under UV‐light illumination. Figure S10 (Supporting Information) shows the time‐resolved response of four GeS sensor devices. Each of the GeS flakes has a different shape and thickness (70–470 nm), resulting in different current levels; however, all sensors exhibited relatively the same shape of time‐resolved current response as in the primary sensor shown in Figure 3d which had a thickness of 170 nm. As most of the analyte‐sensing layer interaction, i.e., physisorption or chemisorption occurs on the exposed surface of the 2D material, variation in flake's thickness and shape does not affect the VOC sensing performance significantly, so the unique time‐resolved current signature is consistent and stable. The limit of detection (LOD) is another important parameter that is defined as the lowest concentration of VOC that can be detected by a gas sensor. The GeS sensor exhibited a LOD of ≈10 ppm for VOCs, which was limited by the testing setup. There are various diseases that could be detected at the ppm level. For example, Zhu et al. proposed a method to detect and distinguish VOCs in a range of a few hundred ppm levels based on plasma‐enhanced IR absorption spectroscopy for early healthcare diagnosis of diseases such as diabetes.^[^ ^11^ ^]^ A comparison of the other VOC sensors based on 2D materials is summarized in Table S1 (Supporting Information). The GeS sensor exhibited higher sensing performance with ultra‐selective response to VOCs compared to the other reported previously 2D material‐based VOC sensor.

## 3 Conclusion

In summary, we demonstrated a new approach to identify VOC species based on the photoactivated time‐resolved electrical response of 2D GeS sensors. The GeS sensor exhibited a time‐resolved electrical signature unique to each VOC during UV excitation. This unique “fingerprint” facilitates discrimination of one VOC from others. A proof‐of‐concept machine learning algorithm was employed to quantify VOCs extracted from their dynamic response via visualization allowing the identification of ethanol, acetone, and 2‐propanol. The proposed method demonstrates 2D GeS as a promising candidate for a selective VOCs sensor with potential applications such as non‐invasive diagnostic screening tools, hazardous environmental monitoring, and process control.

## 4 Experimental Section

### Fabrication of GeS Sensors

Thermally grown SiO_2_ (300 nm in thickness) coated Si substrates (resistivity ≤ 0.005 ohm‐cm) were cleaned in acetone, 2‐propanol, and DI water ultrasonication baths for 10 min each. GeS flakes were mechanically exfoliated from a bulk single crystal (2D Semiconductors) using blue Nitto tape. The tape was then firmly pressed against a SiO_2_/Si substrate and peeled off rapidly, leaving behind multilayer GeS nanoflakes on the substrate. Interdigitated electrodes were formed by photolithography using Microposit S1813 positive photoresist by lift‐off process. The patterned substrates were coated by 10 nm Cr and 60 nm Au deposited by thermal evaporation, and a subsequent lift‐off process was performed in an acetone bath.

### Characterization

The thickness of the GeS flake was measured by atomic force microscopy (AFM, Asylum MFP‐3D). The Raman spectra of samples were obtained using a Renishaw via confocal Raman microscope equipped with a 514 nm continuous‐wave excitation laser. The TEM lift‐out sample was prepared with an FEI (now part of Thermo Fisher) Helios NanoLab 650 FIB/SEM system, using 30 keV Ga ion beams for lifting out and thinning and 16 kV Ga ion beams for final polishing. The SAED, HR‐TEM, HAADF STEM, and EDX images were taken with an FEI (Thermo Fisher) Tecnai Osiris S/TEM system equipped with Bruker SuperX EDX detectors and operated at 200 keV.

### Device Measurements

A lab‐made VOC sensing setup system was used to characterize VOC sensing properties. N_2_ from a liquid nitrogen dewar was used as the carrier gas. An N_2_ line was connected to a gas sampling bulb via a flow controller. A measured volume of liquid VOCs was injected into the gas sampling bulb using a syringe and a heater was used to generate VOCs vapor. The output of the bulb was connected to the sensor probing chamber (HFS350EV‐PB4 Linkam). The concentration of VOCs was calculated by the volume of VOC liquids and the flow of N_2_ gas. The VOC concentration was calculated by a well‐known theoretical formula as follows:^[^ ^59^ ^]^

(1) $$C_{\mathrm{ppm}}=\frac{T\left(K\right)\times \rho \times R\times V_{\mathrm{Liqu}\mathrm{id}}}{M_{\mathrm{Liqu}\mathrm{id}}\times V_{\mathrm{Chamber}}}$$

where *T*(*K*) is the temperature of the inside chamber, *ρ* (g mL^−1^) is the density of VOCs, *R* is the gas constant, *V*_Liquid_ is the volume of injected VOC liquid, *V*_chamber_ is the volume of the chamber, and *M* is the molecular weight of VOC liquid. The sensor devices were mounted into the sealed stainless‐steel probing chamber and contacted using tungsten needle probe tips. The electrical characteristics during VOC measurements were collected using a Keithley 2400 source meter. Current‐voltage measurements of the GeS FET were taken using a Keithley 4200 Semiconductor Characterization System and probe station. A UV LED lamp (365 nm) was fixed above the probing chamber and the UV light was incident directly on the GeS sensor through a window on the top of the probing chamber. The sensor response was measured as Δ*G*/*G_o_* where Δ*G* is the change in conductance due to VOC and *G*_o_ is the original conductance without VOC.

### Implementing Machine Learning

The naïve Bayes classifier, which employs a simple technique to assign class labels to problem instances, was utilized as the classification technique. After the training, classification loss by resubstituting the samples was estimated and the loss was set equal to zero (every sample was classified correctly).

## Conflict of Interest

The authors declare no conflict of interest.

## Author Contributions

A.H. and M.R.M. contributed equally to this work. A.H. and M.R.M. performed all experimental design and fabrication under the supervision of M.M.A. A.H., M.R.M., and M.M.A. co‐wrote the manuscript. K.J. performed implemented machine learning and signal classification analysis. M.F. performed AFM characterization. T.D.S., A.Abnavi, and R.A. participated in data analysis. H.G. performed Raman measurements. A.Askar and F.K. participated in electrical measurements. R.K.N.D.R. and M.M.A. provided project leadership and supervision.

## Supporting information

Supporting Information

## Data Availability

The data that support the findings of this study are available from the corresponding author upon reasonable request.
