# Supplementary material for: Unique Photoactivated Time‐Resolved Response in 2D GeS for Selective Detection of Volatile Organic Compounds
Source: Adv Sci (Weinh). 2023 Jan 19;10(10):2205458. doi: 10.1002/advs.202205458 (PMC10074048; doi:10.1002/advs.202205458)
Supplement: Supplementary file 1 — Supporting Information [file ADVS-10-2205458-s001.pdf]

ADVANCED SCIENCE
Open Access

Supporting Information

for *Adv. Sci.*, DOI 10.1002/advs.202205458

Unique Photoactivated Time-Resolved Response in 2D GeS for Selective Detection of Volatile Organic Compounds

*Mohammad Reza Mohammadzadeh, Amirhossein Hasani, Keyvan Jaferzadeh, Mirette Fawzy, Thushani De Silva, Amin Abnavi, Ribwar Ahmadi, Hamidreza Ghanbari, Abdelrahman Askar, Fahmid Kabir, R.K.N.D. Rajapakse and Michael M. Adachi**

Unique Photoactivated Time-Resolved Response in Two-Dimensional GeS for Selective Detection of Volatile Organic Compounds

Mohammad Reza Mohammadzadeh [+], *Amirhossein Hasani* [+], *Keyvan Jaferzadeh* [b], *Mirette Fawzy* [c], *Thushani De Silva* [a], *Amin Abnavi* [a], *Ribwar Ahmadi* [a], *Hamidreza Ghanbari* [a], *Abdelrahman Askar* [a], *Fahmid Kabir* [a], *R.K.N.D. Rajapakse* [a], *and Michael M. Adachi* [a], *

M.R. Mohammadzadeh, A. Hasani, T.D. Silva, A. Abnavi, R. Ahmadi, H. Ghanbari, A. Askar ,F. Kabir, R.K.N.D. Rajapakse, M.M. Adachi

School of Engineering Science, Simon Fraser University, Burnaby V5A 1S6 British Columbia, Canada.

K. Jaferzadeh

Department of Computer Science and Software Engineering, Concordia University, Montreal, H3G 1M8, Quebec, Canada.

M. Fawzy

Department of Physics, Simon Fraser University, Burnaby V5A 1S6 British Columbia, Canada.

*E-mail: mmadachi@sfu.ca

[+] These authors contributed equally to this work.

KEYWORDS: 2D Materials, VOC detection, GeS, Machine learning, sensors

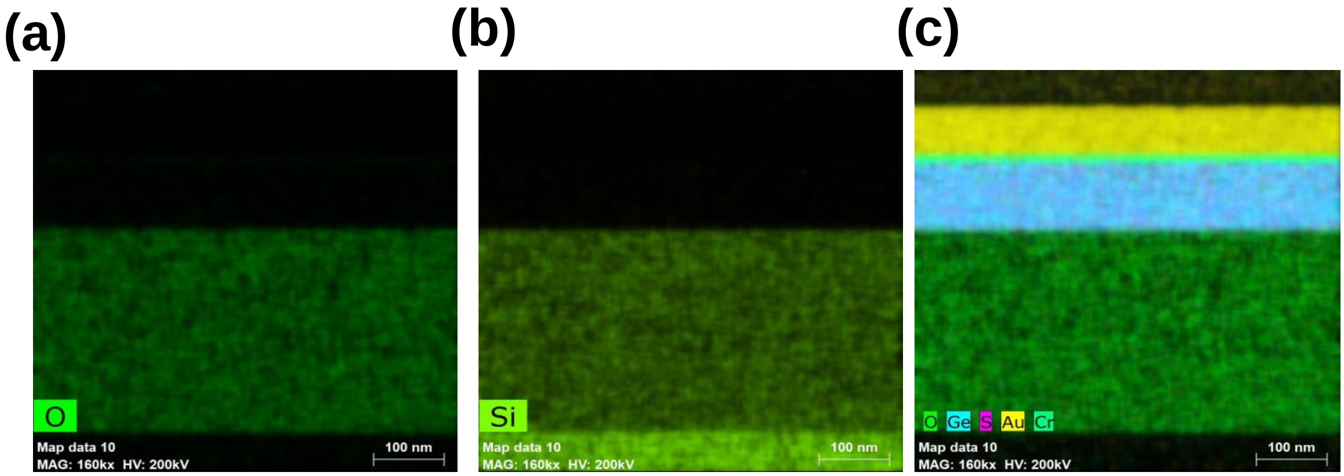

Figure S1 (a) elemental mapping images of O, (b) Si, and (c) cross-section image of the combined element mapping of a layered system.

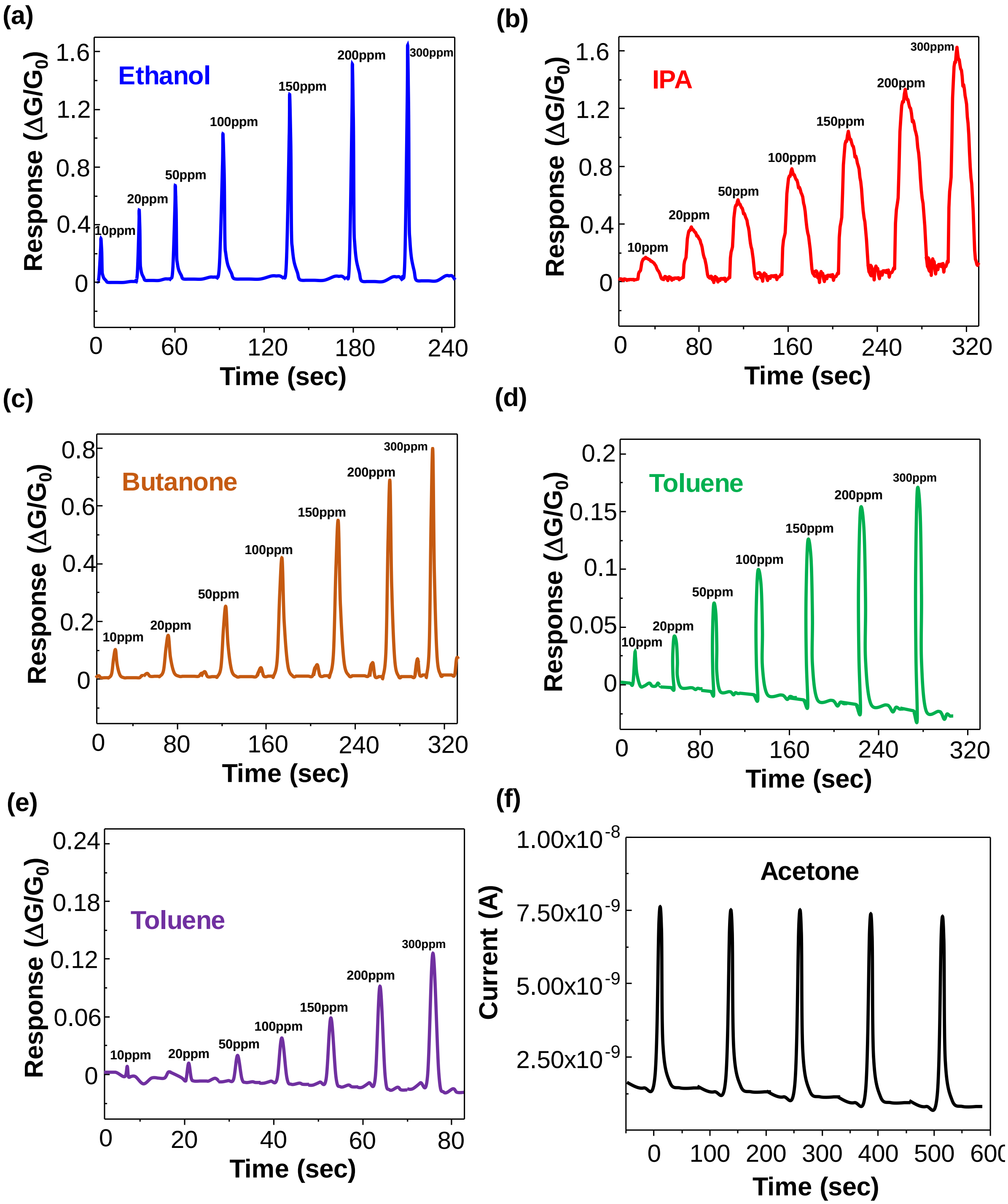

Figure S2 (a) Time-resolved current response of GeS sensor exposed to ethanol, (b) IPA, (c) butanone, (d) toluene, (e) hexane under dark environment, and (f) cyclability test of GeS sensor toward 200 ppm of acetone under dark condition.

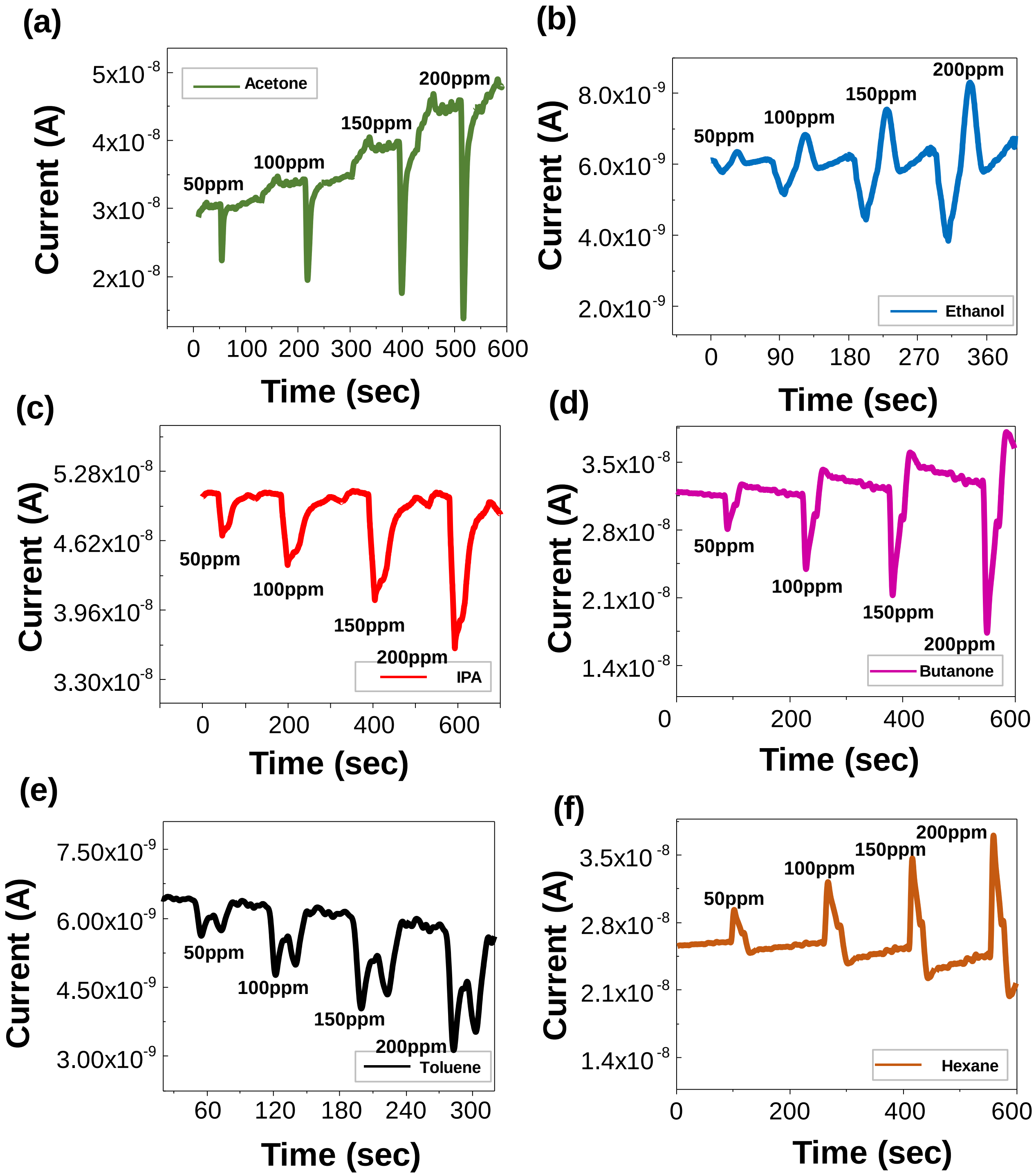

Figure S3 (a) Time-resolved current response of GeS sensor exposed to different concentrations (50,100,150 and 200 ppm) of ethanol, (b) IPA, (c) butanone, (d) toluene, (e) hexane under UV-light illumination.

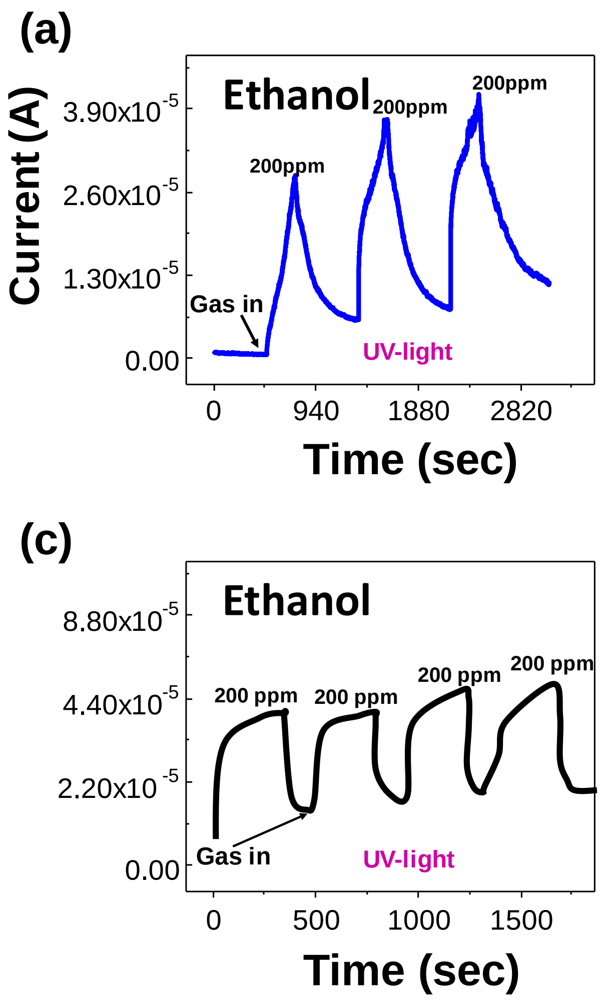

Figure S4 (a) Time-resolved current response of MoS_2 sensor exposed to 200 ppm of ethanol, and (b) IPA under UV-light illumination, (c) time-resolved current response of $MoSe_2$ sensor exposed to 200 ppm of ethanol, and (d) IPA under UV-light illumination.

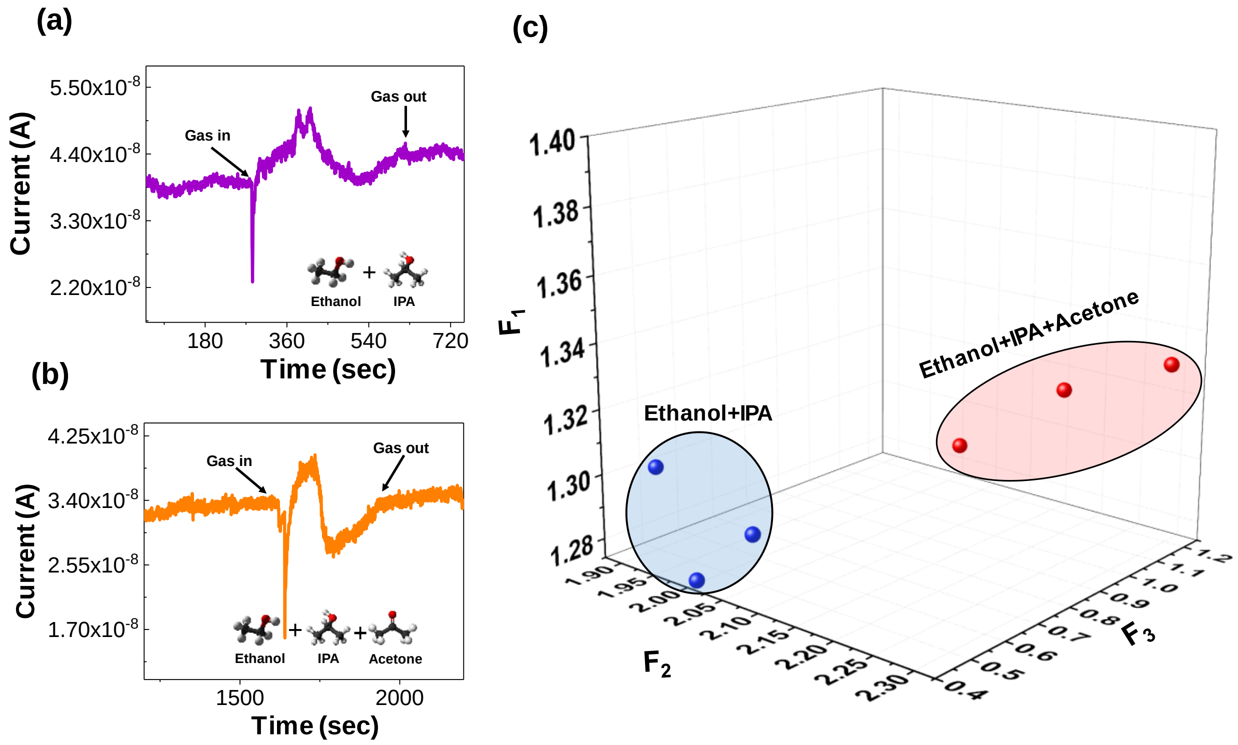

Figure S5 (a) Time-resolved current response of GeS sensor exposed to ethanol and IPA, (b) ethanol, IPA, and acetone mixture, (c) 3D distribution of the three features for the two different VOC mixtures.

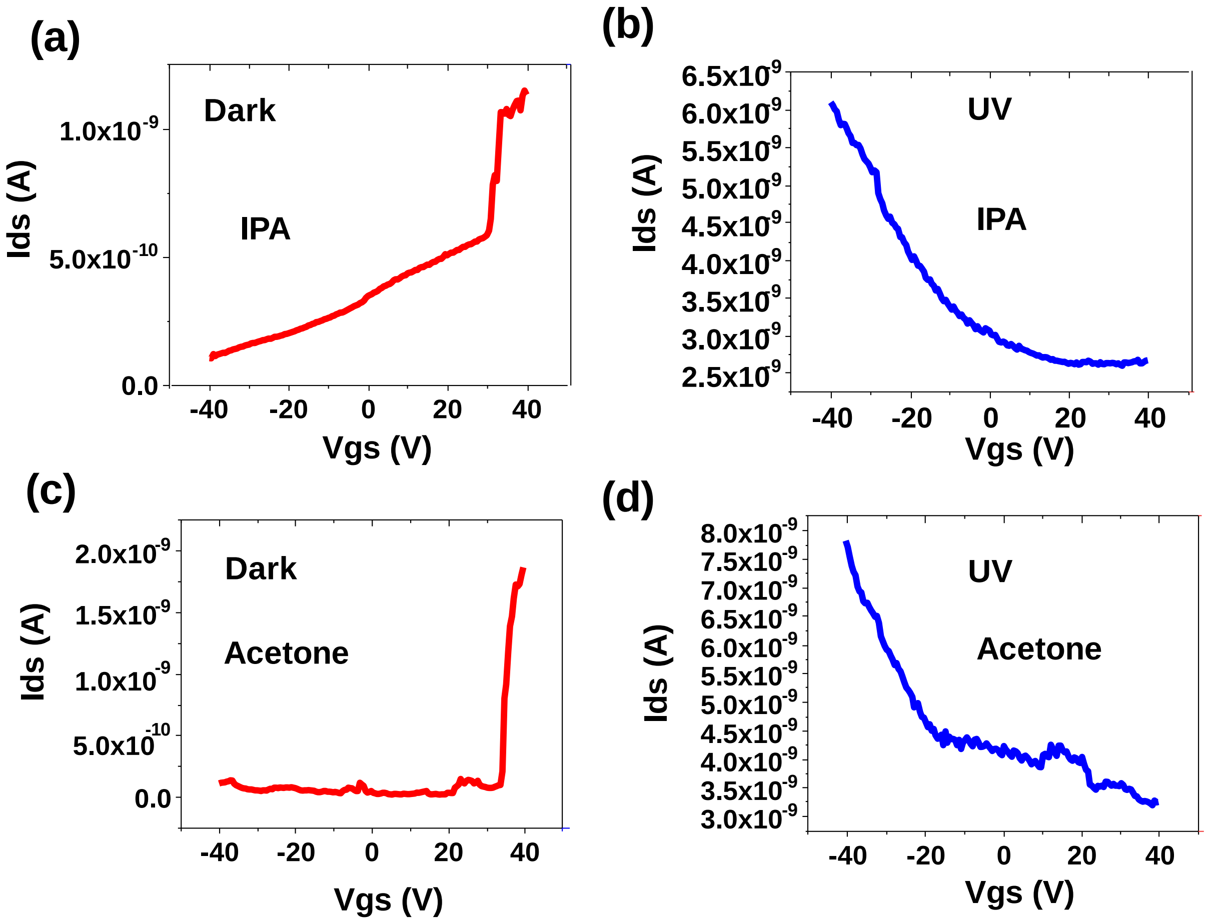

Figure S6 (a) transfer curve of GeS FET exposed to IPA under dark, (b) and UV-light illumination, (c) transfer curve of GeS FET exposed to acetone under dark, (d) and UV-light illumination.

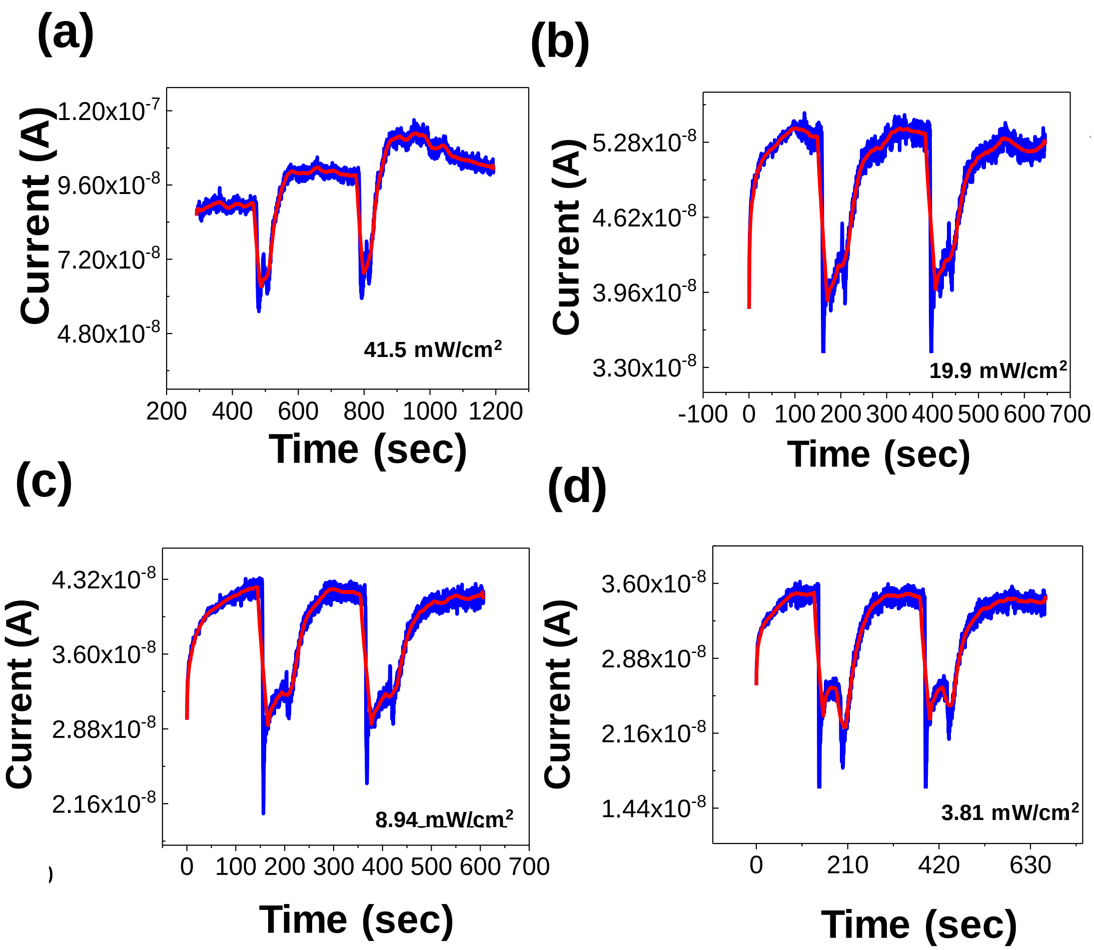

Figure S7 two cycles time-resolved current response of GeS sensor exposed to 200 ppm of IPA under at different UV-ligh powers.

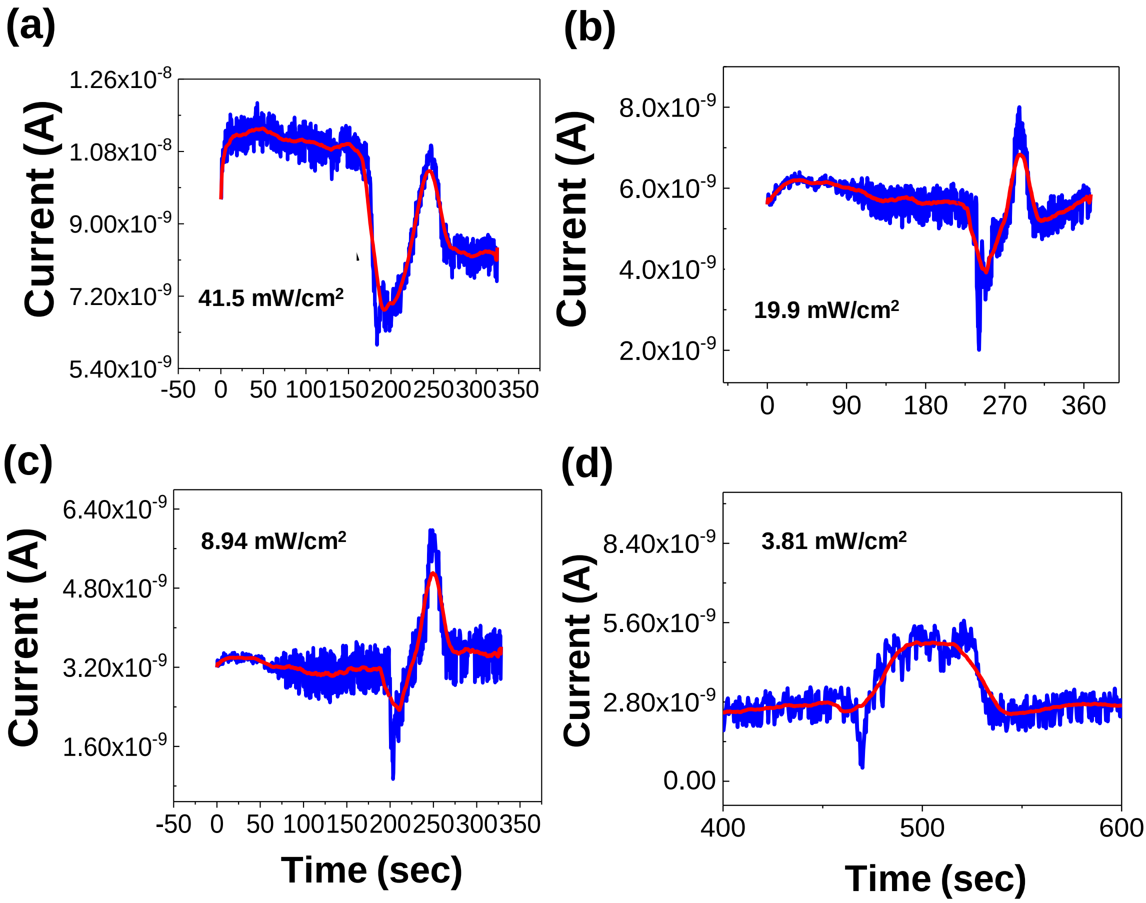

Figure S8 one cycle time-resolved current response of GeS sensor exposed to 200 ppm of ethanol under at different UV-light powers.

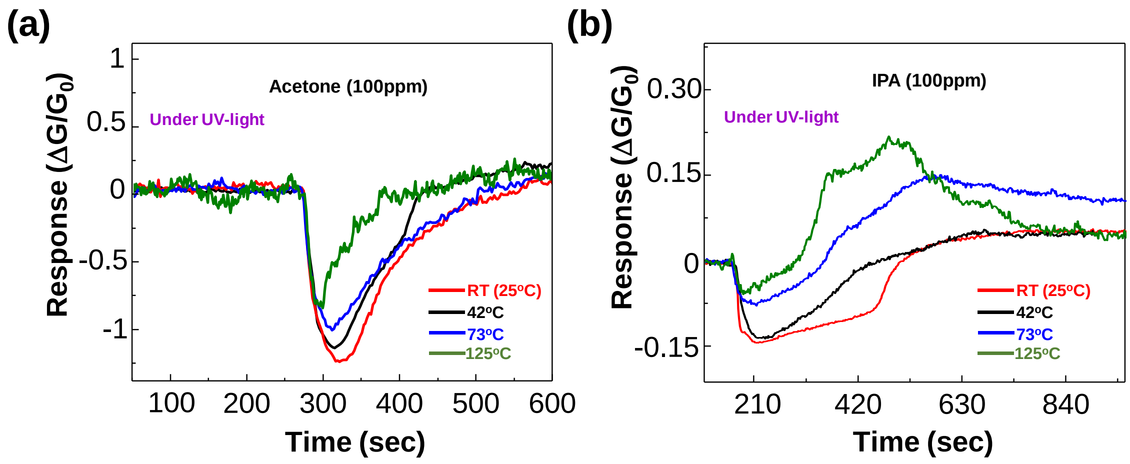

Figure S9 (a) Effect of temperature on the 100 ppm of acetone, and (b) IPA sensing performance.

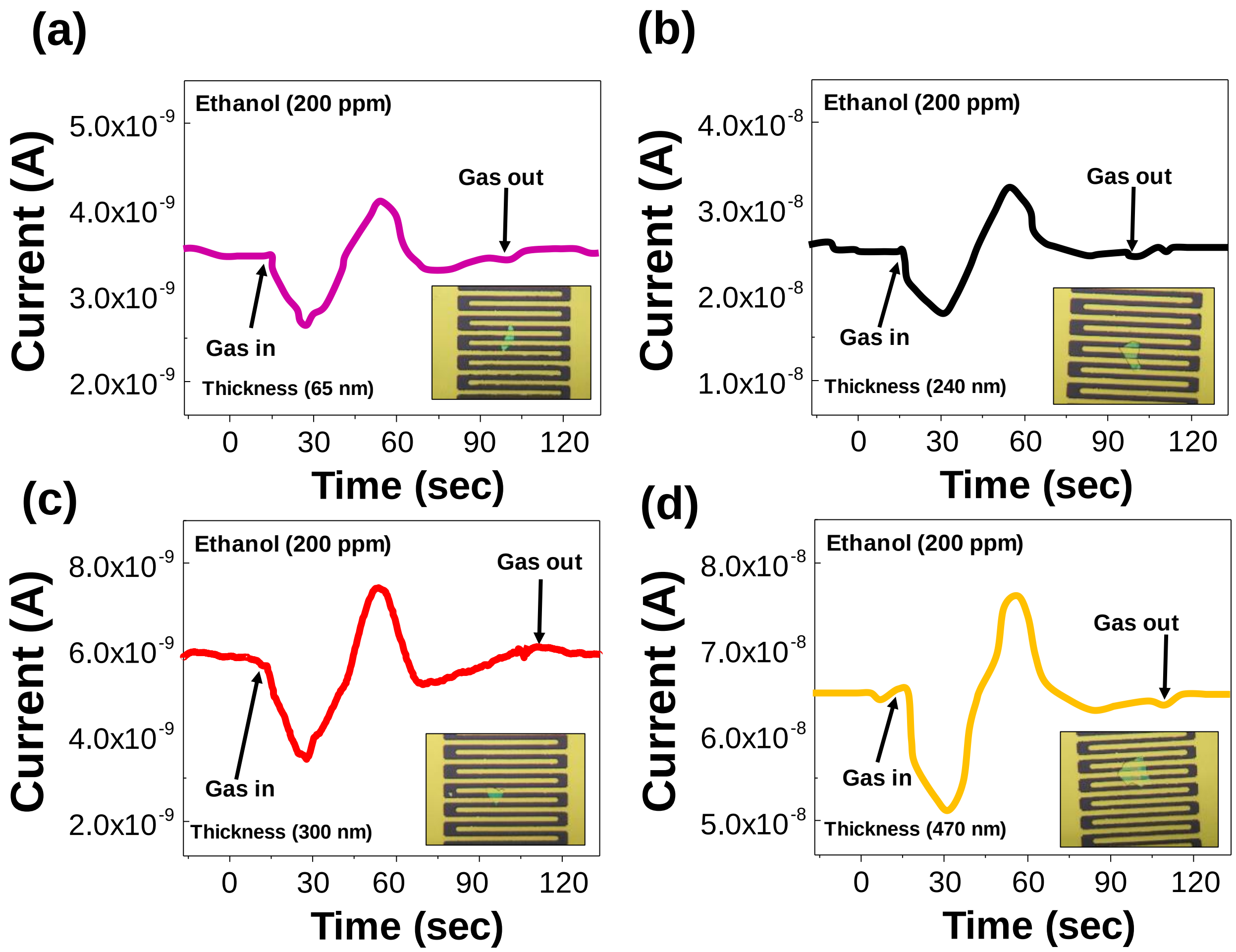

Figure S10 Time-resolved response of the as-fabricated sensor devices toward 200ppm of ethanol under UV-light illumination.

Table S1 A comparison of the VOC sensing performances at room temperature based on GeS

**Sensing material**	**Target VOC**	**Light source (nm)**	**Response/sensitivity**	**Response/recovery time**	**Environment**	**Ref.**
ReS_2	Acetone (100ppm)	650nm	104.8%	-	Air, RH50%	[1]
WSe_2	Acetone (10ppm)	-	~17%	6.5min/43sec	N_2	[2]
$MoTe_2$	Acetone (100ppm)	254nm	~58%	~160sec/~3min	N_2	[3]
WS_2	Ethanol (60ppm)	365nm	R_{air}/Rgas =1.2	252sec/648sec	Air, RH25%	[4]
MoS_2/rGO	Formaldehyde (10ppm)	420nm	64%	17sec/98sec	Air	[5]
Graphene	Acetone (1 ppm)	370nm	1.87%	200sec/5min	N_2	[6]

(our work) and other 2D materials.

$Ti_3C_2T_x$ MXene	Ethanol (10ppm)	390nm	91%	~25sec/~85sec	Air	[7]
Au-doped MoS_2	Acetaldehyde (1000 ppm)	-	6%	~3min/4min	N_2	[8]
GeS	Acetone (100ppm)	Dark, 365nm	262% (dark), 95% (365nm)	8.6sec/13.4sec	Air, N_2, RH40%	This work

References:

[1] A. Zulkefli, B. Mukherjee, R. Sahara, R. Hayakawa, T. Iwasaki, Y. Wakayama, S. Nakaharai, *ACS Applied Materials & Interfaces* **2021**, *13* (36), 43030.
[2] K. Y. Ko, K. Park, S. Lee, Y. Kim, W. J. Woo, D. Kim, J.-G. Song, J. Park, H. Kim, *ACS applied materials & interfaces* **2018**, *10* (28), 23910.
[3] E. Wu, Y. Xie, B. Yuan, D. Hao, C. An, H. Zhang, S. Wu, X. Hu, J. Liu, D. Zhang, *ACS applied materials & interfaces* **2018**, *10* (41), 35664.
[4] D. Gu, X. Li, H. Wang, M. Li, Y. Xi, Y. Chen, J. Wang, M. N. Rumyntseva, A. M. Gaskov, *Sensors and Actuators B: Chemical* **2018**, *256*, 992.
[5] J. Wang, H. Deng, X. Li, C. Yang, Y. Xia, *Sensors and Actuators B: Chemical* **2020**, *304*, 127317.
[6] C.-M. Yang, T.-C. Chen, Y.-C. Yang, M. Meyyappan, C.-S. Lai, *Sensors and Actuators B: Chemical* **2017**, *253*, 77.
[7] M. Hou, J. Gao, L. Yang, S. Guo, T. Hu, Y. Li, *Applied Surface Science* **2021**, *535*, 147666.
[8] S.-Y. Cho, H.-J. Koh, H.-W. Yoo, J.-S. Kim, H.-T. Jung, *ACS sensors* **2017**, *2* (1), 183.
